# PARP inhibitor olaparib sensitizes cholangiocarcinoma cells to radiation

**DOI:** 10.1002/cam4.1318

**Published:** 2018-02-26

**Authors:** Yize Mao, Xin Huang, Zeyu Shuang, Guohe Lin, Jun Wang, Fangting Duan, Jianlin Chen, Shengping Li

**Affiliations:** ^1^ State Key Laboratory of Oncology in South China Collaborative Innovation Center for Cancer Medicine Sun Yat‐sen University Cancer Center Guangzhou 510060 China; ^2^ Department of Hepatobiliary Oncology Sun Yat‐sen University Cancer Center Guangzhou 510060 China; ^3^ Department of Breast Oncology Sun Yat‐sen University Cancer Center Guangzhou 510060 China; ^4^ Department of Experimental Research Sun Yat‐sen University Cancer Center Guangzhou 510060 China; ^5^ Department of Ultrasound Sun Yat‐sen University Cancer Center Guangzhou 510060 China

**Keywords:** Cholangiocarcinoma, olaparib, poly(ADP‐ribose) polymerase, radiation

## Abstract

Cholangiocarcinoma (CCA) is a highly malignant tumor with resistance to radiotherapy alone. Olaparib, a highly potent poly(ADP‐ribose) polymerase (PARP) inhibitor, has been shown to sensitize many types of tumor to radiotherapy. However, the effect of olaparib, either as monotherapy or as combination therapy with radiotherapy, on CCA is not known, and our study aimed to explore this. To assess radiosensitization in three CCA cell lines (QBC939, HuH28 and TFK‐1), viability and clonogenic assays were conducted. The absorbed radiation doses were 0 Gy, 2 Gy, 4 Gy, and 6 Gy; olaparib concentrations were 0 nmol/L, 1 nmol/L, 10 nmol/L, 100 nmol/L, 1000 nmol/L, 2500 nmol/L, 5000 nmol/L and 10 000 nmol/L. The mechanism of olaparib radiosensitization was explored by Western blotting. Immunofluorescence staining and flow cytometry were conducted to explore DNA damage and apoptosis. The radiosensitivity of CCA cells was enhanced by olaparib, which alone had little effect on the CCA cell lines without BRCA mutations. The degree of radiosensitization increased with increasing doses of olaparib by viability and clonogenic assays *in vitro*. Olaparib was able to enhance the effect of radiation by inhibiting PARP1, inducing DNA lesions and apoptosis. These findings emphasize the role of olaparib in the radiosensitization of CCA cells.

## Introduction

Cholangiocarcinoma (CCA) is the second most common primary liver cancer originating from the epithelium of the bile ducts and has generally shown increasing incidence in recent years, with exceptions for certain geographic and genetic factors 1, 2. Surgical resection with histologically negative resection margins was thought to be the only potentially curative option for patients with CCA, because of its relative resistance to radiotherapy and chemotherapy 3, 4, 5. However, only a few cases are suitable for surgery at diagnosis 6, 7. In addition, recurrences in the hepatic hilar region remain a difficulty after surgery, especially after cholangiojejunostomy 8. As a result, other conservative therapeutic modalities, such as endoscopic or percutaneous biliary drainage, offering a purely palliative role to improve the quality of life, are often recommended 9. The results of these clinical treatments for unresectable CCA are dismal, with 5‐year overall survival rate lower than 10% 10, and new strategies for those patients not eligible for surgery are urgently needed.

A number of strategies that combine radiotherapy with novel targeted agents to enhance the efficacy of the radiotherapy have been tested 11, 12, 13, 14, 15. Olaparib, a highly selective potent poly(ADP‐ribose) polymerase (PARP) inhibitor, that inhibits base excision repair and single‐strand DNA break (SSB) repair, has recently been approved for therapy of ovarian and breast cancer 16, 17, 18. Preclinical studies have shown that PARP inhibition produces radiosensitization in multiple cancers, especially those with BRCA1 and BRCA2 mutations, which lead to homologous recombination deficiency 11, 19, 20.

Recent studies showed that about 40% of CCA contains the genetic alterations in potential therapeutic targets, such as FGFR, IDH1 and BRCA1/2 21. Moreover, studies have shown a low prevalence of BRCA1/2 mutations in CCA (≤4%) 21, 22, while another study revealed that the frequency of BRCA1 and BRCA2 mutations was 17 and 0% in extrahepatic CCA cases, respectively 23. In addition, other studies showed that the germline BRCA1/2 mutations are not necessary for patients to derive benefits from PARP inhibitors 24, 25, 26.

However, whether olaparib could enhance the radiosensitivity of CCA is not clear, so we aimed to detect BRCA1/2 mutations in CCA cells and evaluate the influence of olaparib doses with different radiation doses on the growth of CCA cells. We hypothesized that olaparib and radiotherapy together could present a novel and effective therapeutic strategy for patients with CCA.

## Methods

### Cell culture

The human CCA cell lines TFK‐1 (DSMZ, Braunschweig, Germany), QBC939 (Cell Bank of Chinese Academy of Sciences, Shanghai, China) and HuH28 (RIKEN, Saitama, Japan) were cultured in RPMI‐1640 (Invitrogen Corp., USA) supplemented with 10% heat‐inactivated fetal bovine serum (Gibco‐BRL, Carlsbad, California, USA), as recommended by the supplier, at 37°C in a humidified atmosphere under 5% CO_2_.

### DNA extraction and sequencing

Genomic DNA was extracted from cells using QIAamp DNA mini kit (Qiagen, Germany). Double‐stranded DNA was quantified by a Picogreen fluorescence assay using Qubit3.0 (Life Technologies, USA).

Targeted genomic areas were amplified by polymerase chain reaction (PCR) from 30 ng of genomic DNA. PCR reactions were prepared using the Ion AmpliSeq Library Kit 2.0 and Ion AmpliSeq^™^ BRCA1 and BRCA2 Panel according to the manufacturer's instructions. The resulting amplicons were treated with FuPa Reagent to partially digest the primers and then ligated to Ion P1 and IonXpress Barcode adapters (all Life Technologies, USA). Barcoded libraries were purified using AgencourtAMPure^™^ XP Reagent (Beckman Coulter, USA) and equalized to 100 pM following the recommended protocol.

All procedures for emulsion PCR and next‐generation sequencing were performed with Ion Torrent equipment and kits (all Life Technologies, USA), according to the manufacturer's specifications: Template‐positive ion sphere particles (ISPs) containing clonally amplified DNA were generated from emulsion PCR using the Ion PI^™^ Hi‐Q^™^ OT2 200 kit with the Ion OneTouch 2 instrument. Enrichment of template‐positive ISPs was performed with an Ion OneTouch ES. Sequencing of enriched templates bound to ionospheres was performed on an Ion Proton Sequencer using the Ion PI Hi‐Q^™^ Sequencing 200 kit and Ion PI chip.

Data from the sequencing runs were processed initially using the Ion Torrent platform‐specific pipeline software (Torrent Suite Version 4.4) to generate sequence reads, separate barcoded reads, trim adapter sequences, and filter and remove poor signal‐profile reads. Initial variant calling from the Ion AmpliSeq sequencing data was generated using Torrent Suite Software v4.4 with a plugin “variant caller” program.

Variants found in the Ion Torrent Variant Caller were further analyzed to determine the likelihood that the variant was deleterious.

### Radiation

Cells were exposed to single radiation doses (0, 2, 4 and 6 Gy) at a dose rate of 1.2 Gy/min using the RS2000 (Rad Source Technologies, USA).

### Assays used

Cell viability was assessed by plating proliferating cells onto a 96‐well culture plate and treating them with 0, 1, 10, 100, 1000, 2500, 5000, or 10, 000 nmol/L of olaparib (Selleck Chemicals, Munich, Germany) in pure dimethylsulfoxide (DMSO) for 1 h prior to irradiation. Ongoing culture then continued in the presence of olaparib. Four days after irradiation, Cell Counting Kit‐8 (CCK8; Dojindo, Kumamoto, Japan) assays were performed, with 10 *μ*L CCK‐8 solution being added into each well. After incubation at 37°C with 5% CO_2_ for 2.5 h, the optical density was read with a microplate reader (Bio‐Rad, La Jolla, USA) at 450 nm. The data were represented as “viability”.

Clonogenic survival was assessed by plating 500 proliferating cells onto a 6‐well culture plate and irradiating them after a 1‐h olaparib pre‐incubation. The cells were then cultured in the continuous presence of 1 *μ*mol/L olaparib, as described previously, until colony formation (2 weeks). Then, the cell clones were washed twice with PBS, fixed in methanol for 15 min, stained with crystal violet for 15 min at room temperature and counted.

Western blot analysis was conducted as described previously 27, using the following antibodies, including anti‐GAPDH(1:5000, 60004‐1‐Ig; Proteintech, USA), anti‐PARP1 (1:1000, ab32138; Abcam, UK), and anti‐PAR (1:500, ab14459; Abcam, UK).

For immunofluorescence staining, cells were planted on glass cover‐slips onto 24‐well plates. Then, those cells were treated with 1 *μ*mol/L olaparib or not. One hour later, those tumor cells were irradiated (4 Gy) or not. Eight hours later, those cells were washed with PBS and fixed with freshly prepared 4% paraformaldehyde at room temperature for 20 min. Then, 0.5% Triton X‐100 (TX)/PBS was used to permeabilize the tumor cells for 20 min. Then, the tumor cells were washed thrice with PBS and blocked by 5% BSA/0.1% TX/PBS for 30 min at room temperature. Then, the tumor cells were incubated with *γ*H2AX antibody (1:100 dilution; Millipore, USA) overnight at 4°C. After washing with 0.01% Triton X‐100 (TX)/PBS thrice, the tumor cells were incubated for 1 h with secondary antibody (goat anti‐rabbit Alexa 488‐conjugated antibody at 1:500 dilution) at 37°C. Nuclei were stained with 4′, 6‐diamidino‐2‐phenylindole (1:1000 dilution; Molecular probes, USA). Images were viewed and assessed using a scanning confocal microscope (Fluoview FV1000, OLYMPUS, Tokyo, Japan) and analyzed using FV10‐ASW (viewer 4.0). Cells with *γ*H2AX foci not less than 10 were considered as positive 28.

For apoptosis analysis, tumor cells were planted into six‐well plates and treated or not treated with 1 *μ*mol/L olaparib. After 1 h, those tumor cells were irradiated (4 Gy) or not irradiated. Then, 72 h later, the tumor cells were harvested. About 10^6^ cells were stained with annexin V‐fluorescein isothiocyanate (FITC; 10uL/mL; KeyGEN, Nanjing, China) and propidium iodide (PI; KeyGEN) for 10 min at 4°C. Finally, the percentage of apoptotic cells was measured by flow cytometry (CytoFLEX, Beckman Coulter, USA) and analyzed by Kaluza (Beckman Coulter, USA).

### Data analysis and statistics

All the experiments were repeated independently three times. Cell viability was normalized to either nonirradiated controls or DMSO‐treated controls as indicated. The viability curves were fitted using the linear quadratic (LQ) model (Viability = c*exp[−*αd*−*βd* ^2^], where *d* = radiation dose; or Viability = c*exp[−*αd*−β*log_10_ *d* ^2^], where *d* = dose of olaparib) and the normalized viability curves using the (LQ) model (Viability = exp[−*αd*−*βd* ^2^], where *d* = radiation dose; or Viability = exp[−*αd*−β*log_10_ *d* ^2^], where *d* = dose of olaparib).

The radiation dose enhancement factor (DEF) of olaparib is the ratio of the radiation doses resulting in 50% survival (DEF_50_) comparing control samples to olaparib‐treated samples. The area under the curve (AUC) of the normalized olaparib effect curve with doses of radiation ranging from 0 to 6 Gy (AUC_0–6 Gy_) was calculated.

Clonogenic survival was the ratio of the number of cell colonies with olaparib treatment compared with DMSO treatment only at each radiation dose. The relative viability was the ratio of the viability at different doses of olaparib and radiation compared to DMSO‐treated samples without radiation. The relative survival fraction (SF) was the ratio of colony numbers for the cells with each treatment compared with the DMSO‐treated cells without radiation.

Python software (version 3.5.1) was used to fit the curve and calculate the value of DEF_50_ and AUC_0–6 Gy_. Statistical analyses were performed using two‐sided Student's *t*‐test (IBM SPSS Statistics software, Version 20.0; SPSS, Inc.). Continuous variables were expressed as mean ± standard deviation (SD). *P* < 0.05 was considered significant.

## Results

### Loss of function mutations of BRCA1/2 were not found in QBC939 and TFK‐1 cell lines

It has been demonstrated that there are no loss of function mutations of BRCA1/2 in HuH28 (https://portals.broadinstitute.org/ccle/page?cell_line=HUH28_BILIARY_TRACT) in the Cancer Cell Line Encyclopedia (CCLE) database. Through next‐generation sequencing, we found QBC939 and TFK‐1 cell lines did not have loss of function variants in BRCA1 and BRCA2 (Table S1).

### Intrinsic sensitivity to olaparib and radiation of CCA cells

**Figure 1:**
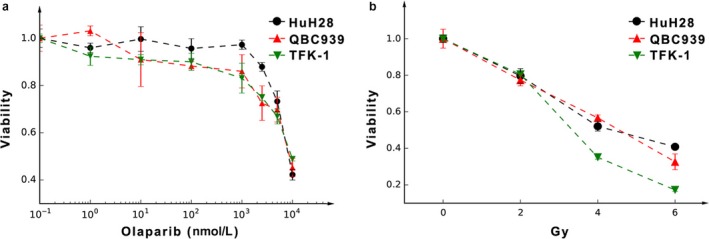
The sensitivity of the three CCA cell lines to radiation or olaparib as determined by viability assays. (a) Growth inhibition after continuous olaparib exposure, with IC_50_ close to 10 *μ*mol/L, the concentration of cells treated with DMSO only (presented as 10^−1^ nmol/L) was taken as the control. (b) Growth inhibition after radiation. Data are presented as the mean ± standard deviation (SD).

To research the radiosensitization effect of olaparib, we must first have a clear understanding of the effect of olaparib or radiotherapy alone on the growth of CCA cells. Olaparib as a single agent is widely used in BRCA1 or BRCA2 mutant tumor cells, but had less effect in CCA cells without BRCA1 or BRCA2 mutations, with a half maximal inhibitory concentration (IC_50_) close to 10 *μ*mol/L (Fig. 1a). Hence, olaparib alone had no obvious effect in killing tumor cells without BRCA1/2 mutations, unless it was used at high concentrations.

With an increasing dose of radiation, the viability of these three types of CCA cells decreased. At 4 and 6 Gy, the viability of TFK‐1 was lower than that of QBC939 and HuH28 (Fig. 1b).

### Olaparib sensitizes CCA cells to radiation

**Figure 2:**
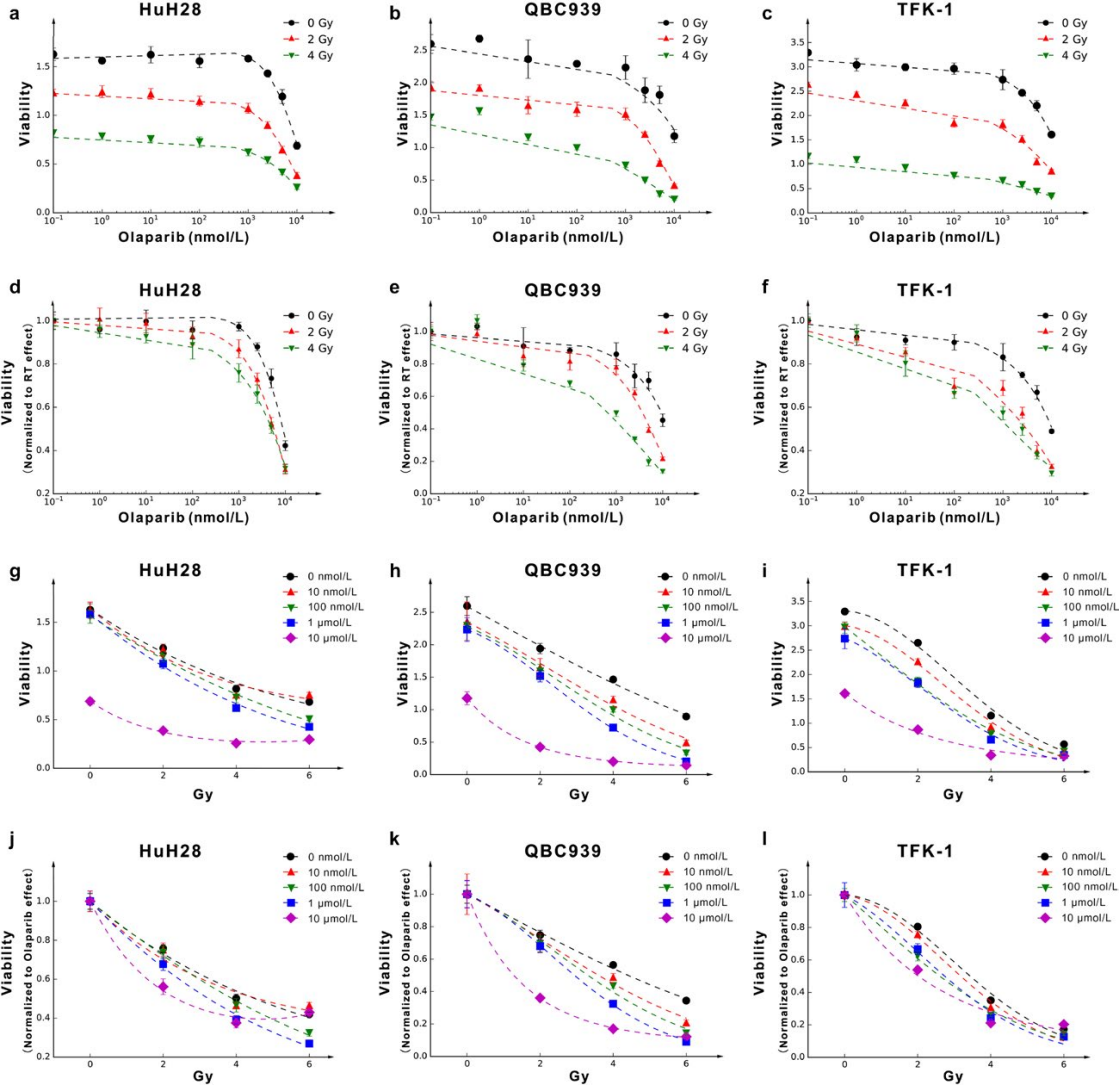
The radiosensitization effect of olaparib in different cell lines. (a–c) Survival in HuH28, QBC939 and TFK‐1 cells after radiation and continuous olaparib exposure as determined by viability assays, with the *X*‐axis denoting the olaparib dose. (d–f) The viability is normalized to nonirradiated values at different olaparib doses. (g–i) Survival in HuH28, QBC939 and TFK‐1 cells after radiation and continuous olaparib exposure as determined by viability assays, with the *X*‐axis denoting the radiation dose. (j–l) The viability is normalized to DMSO‐only (presented as 10^−1^ nmol/L) treatment values at different radiation doses.

Our data show that radiosensitization differs among the various cell lines (Fig. 2). The curves of viability after normalization for the radiation effect alone differed for the differing doses of radiation (Fig. 2a–c) and showed that olaparib enhanced radiosensitivity. Moreover, cell viability decreased steadily with increasing doses of olaparib for each dose of radiation. In general, radiosensitization became apparent with an olaparib concentration of 1 *μ*mol/L, especially at 4 Gy, in each cell line (Fig. 2d–f).

The curves of viability after normalization for the olaparib effect alone differed for the differing doses of olaparib (Fig. 2g–i) and showed that irradiation enhanced the cytotoxicity of olaparib. Moreover, the viability decreased rapidly with increasing doses of radiation in each dose of olaparib (Fig. 2j–l).

This outcome is clinically meaningful, as with the side effects of olaparib, the maximum clinical dose cannot be high and 1 *μ*mol/L is appropriate. This suggests that olaparib may be suitable to act as a radiosensitizer in clinical practice.

### The degree of radiosensitization depends on the types of cell lines and reagent dose of olaparib

**Figure 3:**
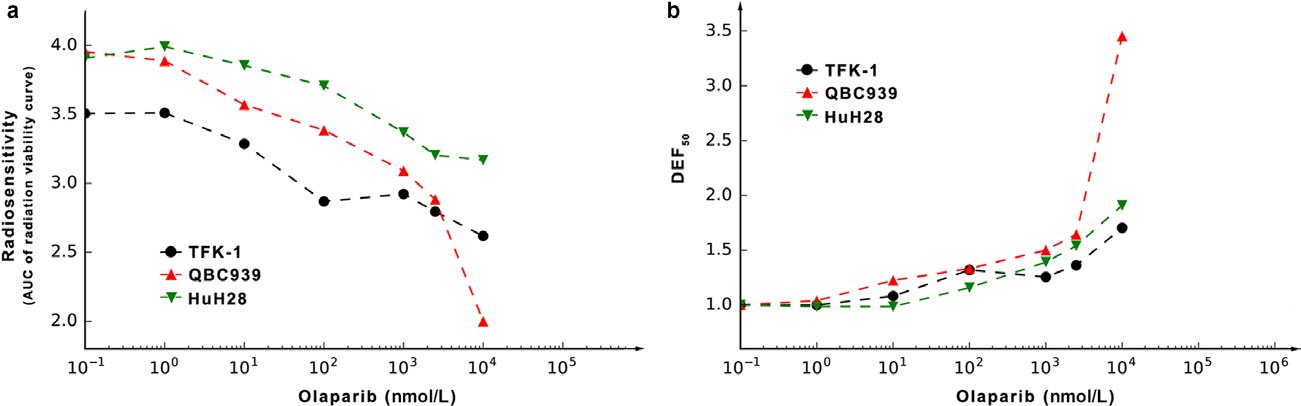
The specific radiosensitization parameters of olaparib. (a) The AUC_0–6 Gy_ was calculated by linear quadratic fit, and the AUC_0‐6 Gy_ at 10 *μ*mol/L was 2.00, 3.17 and 2.62 in QBC939, HuH28 and TFK‐1, respectively. (b) The DEF_50_ was calculated by linear quadratic fit, and the DEF_50_ at 10 *μ*mol/L was 3.45, 1.91 and 1.70 in QBC939, HuH28 and TFK‐1, respectively. AUC_0–6 Gy_ and DEF_50_ reflect the radiosensitization by olaparib for corresponding cells.

To compare the radiosensitization effect of olaparib on different cell lines, as described previously, we introduced the concepts of AUC_0–6 Gy_ and DEF_50_ 11. As shown in Figure 3a, the AUC_0–6 Gy_ value reflected the integrated radiosensitivity of each cell line, including the intrinsic radiosensitivity and radiosensitization effect of olaparib. When olaparib was at 0 nmol/L (shown as 10^−1^ nmol/L), the AUC_0–6 Gy_ value reflected the intrinsic radiosensitivity of each cell line. A lower value indicates greater sensitivity to radiation, so it can be seen that TFK‐1 was the most intrinsically sensitive to radiation. Radiosensitivity increased in each cell line with increasing olaparib concentration.

The DEF_50_ also reflects radiosensitivity, with the value at each concentration of olaparib representing the extent of the decrease in the radiation dose required to achieve the death of 50% of cells compared with radiation alone. As shown in Figure 3b, the degree of radiosensitization became increasingly evident with olaparib concentrations ranging from 0.1 to 10 *μ*mol/L. At 1 *μ*mol/L, the DEF_50_ for the three cell lines QBC939, TFK‐1 and HuH28 was approximately 1.50, 1.26 and 1.39, respectively, indicating a greater radiosensitization effect for olaparib in QBC939 cells. In addition, increasing doses of olaparib produced increased DEF_50_ levels, which showed that radiosensitization depends on the olaparib dose.

### The outcome of the clonogenic survival assay corresponded with our hypothesis

**Figure 4:**
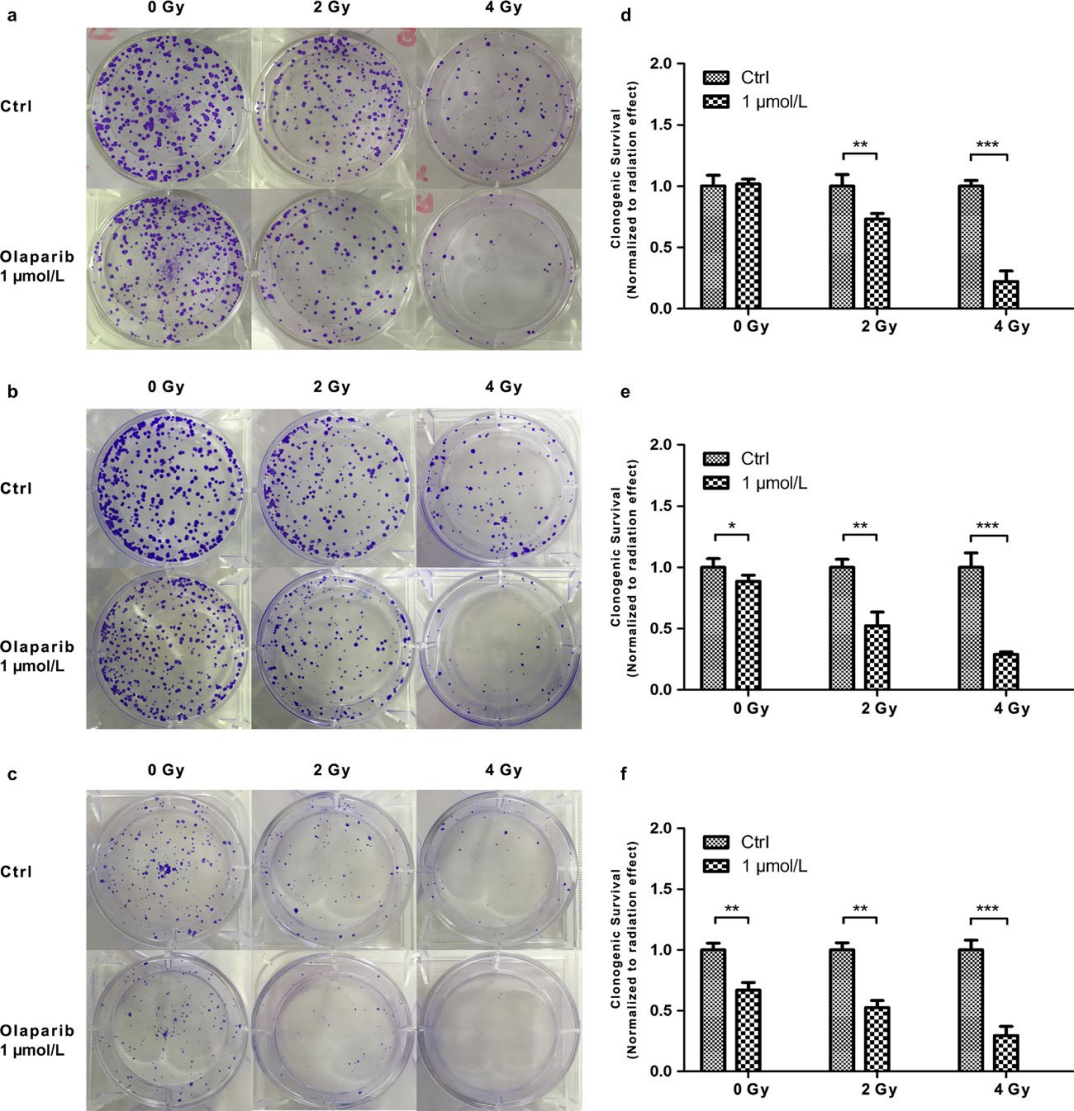
The combination effect of olaparib and radiation for the three CCA cell lines as determined by clonogenic survival assays. (a‐c) Clonogenic survival assay showing the effect of olaparib in each cell line:(a) QBC939; (b) HuH28; (c) TFK‐1. (d–f) Clonogenic survival normalized to the nonirradiated values at 1 *μ*mol/L olaparib for each cell line: (d) QBC939; (e) HuH28; (f) TFK‐1. *0.01 < *P* < 0.05; **0.001 < *P* < 0.01; ***P < 0.001.

From the results stated above, we demonstrated that 1 *μ*mol/L olaparib was appropriate for radiosensitization, so this dose was used to explore the effect of olaparib on clonogenic survival. Consistent with our previous results, the clonogenic survival of each cell line declined with increasing irradiation (Fig. 4). However, treatment with olaparib led to further reductions in clonogenic survival (Fig. 4). These data also showed that olaparib could enhance the radiosensitization of each cell line and that this effect was dependent on the radiotherapy dose.

### Heat maps showing the landscape of the combined effect of olaparib and radiation

**Figure 5:**
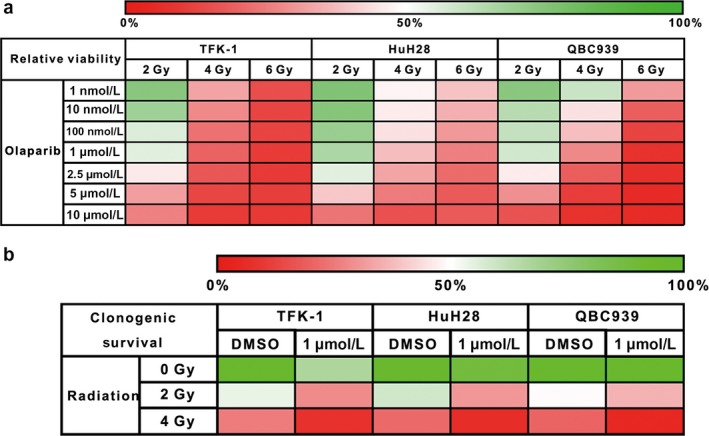
Heat maps showing the landscape of the combination effect of olaparib and radiation. (a) Cell viability assays using the viability of the nonradiated cells with DMSO alone as comparators. (b) Clonogenic survival assays using the SFs of the nonradiated cells with DMSO alone as comparators.

Until now, we have focused only on the radiosensitization effect of olaparib, regardless of its own effect, because the outcome was standardized by the viability of the control sample with 0 nmol/L olaparib or 0 Gy. We produced a heat map taking the combined effect of olaparib and radiation into consideration (Fig. 5a). From this, it can be seen that the killing effect of combination therapy is greatest among the TFK‐1 cell line, confirming our previous result showing it to be more sensitive to radiation.

In accordance with the outcome of the clonogenic survival assay stated above, the heat map of clonogenic survival also showed that the clonogenic survival of each cell line declined rapidly with increasing radiation dose (Fig. 5b).

### Expression and activity of PARP1 under different circumstances

**Figure 6:**
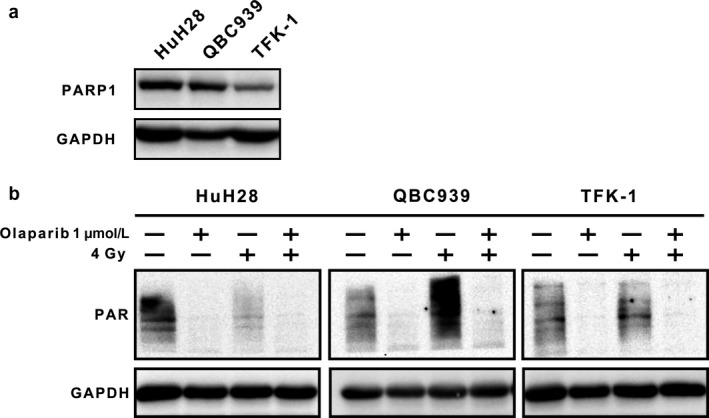
PARP1 expression and activity under different circumstances, with or without radiation. (a) Western blot showing the expression of PARP1 in the three cell lines. (b) Western blot showing the activity of PARP1 by detecting the enzyme product PAR, under different groups; GAPDH was regarded as a loading control.

We found that the TFK‐1 cell line, which is the most sensitive to radiation, expressed the lowest PARP1 (Fig. 6a). At 1 *μ*mol/L concentration, the PAPR1 was extremely suppressed. In addition, at a radiation exposure of 4 Gy, QBC939 presented the biggest increase in the expression of poly(adenosine diphosphate‐ribose) (PAR), the product of PARP1, comparing to the baseline in the corresponding control group (Fig. 6b). This could explain the highest DEF_50_ of QBC939, comparing with TFK‐1 and HuH28, meaning that olaparib exerts the biggest influence on the radiosensitivity of QBC939.

### Combination of olaparib and radiation induces *γ*H2AX foci formation and cell apoptosis

**Figure 7:**
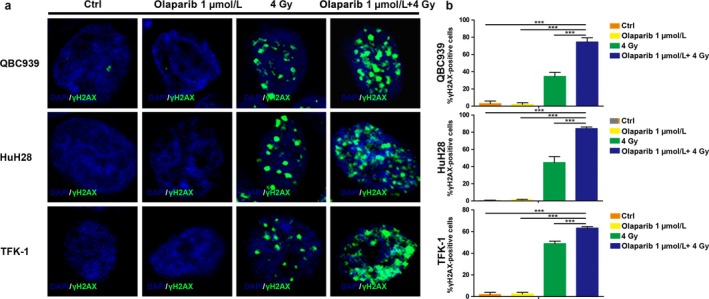
Combination of olaparib and radiation induces *γ*H2AX foci formation. (a) Immunofluorescence staining showing the *γ*H2AX foci in different groups for different cell lines; (b) quantification of the *γ*H2AX foci formation, comparing the number of cells positive for *γ*H2AX foci, with that of all the cells of three independent experiments. ***P < 0.001.

**Figure 8:**
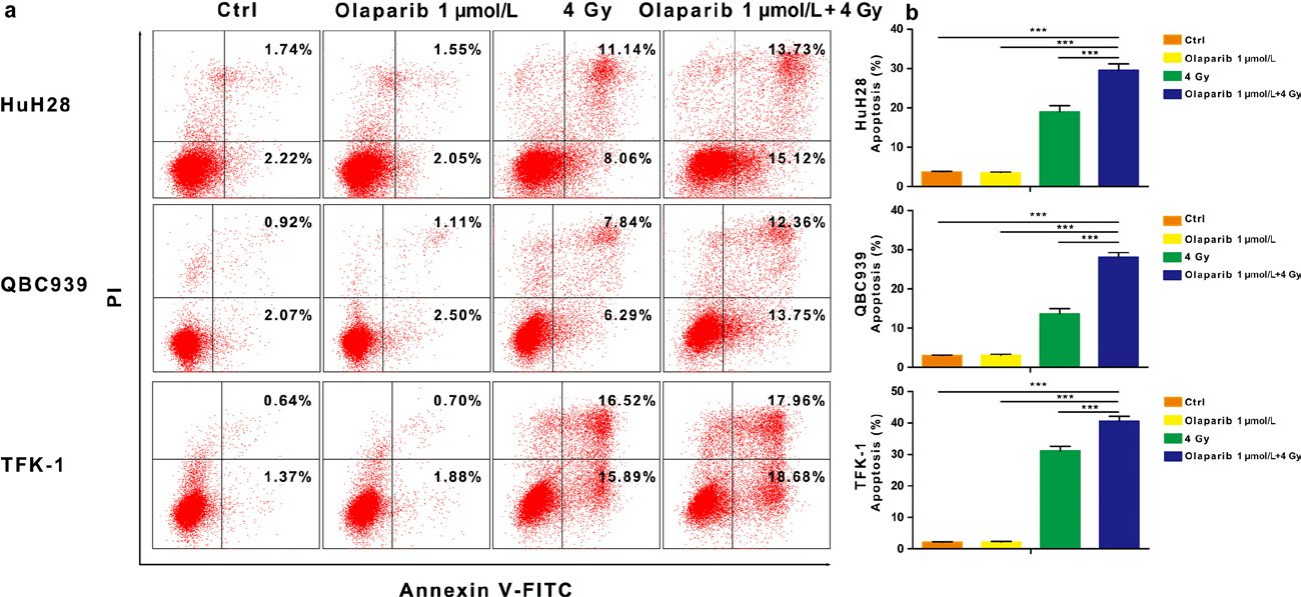
Combination of olaparib and radiation induces apoptosis (a) Flow cytometry showing early and late apoptosis in different groups for different cell lines. (b) Quantification of apoptosis, comparing the number of cells which are Annexin V(+)/PI(−) or Annexin V(+)/PI(+) to that of all the cells of three independent experiments. ***P < 0.001.

An increased level of *γ*‐H2AX is considered to be a marker of DNA damage 29. Hence, we analyzed *γ*H2AX foci in cells treated with 1 *μ*mol/L olaparib(±) and radiation(±). As shown in Figure 7a–b, there was no difference between the tumor cells treated with olaparib and control cells, which suggested that treatment with olaparib alone did not induce DSBs. However, olaparib treatment could enhance the degree of DNA damage caused by radiation (*P* < 0.001). Moreover, we found that apoptosis was highest in the radiation and olaparib combination group than in other groups (*P* < 0.001) (Fig. 8a–b). In total, these data indicated that a combination of olaparib and radiation treatment increased accumulation of unrepaired DSBs in CCA cells and caused apoptosis.

## Discussion

In this study, we discuss the preclinical effect of the PARP inhibitor olaparib on CCA cell growth and its radiosensitivity. As a single agent, olaparib has little effect on the growth of CCA cells, which was consistent with our negative next‐generation sequencing for BRCA1/2 mutations. Intriguingly, when combined with radiation, olaparib showed significant radiosensitization effects, the extent of which was dependent on the type of cell line and olaparib dose.

The PARPs are a marvelous family of multifunctional enzymes, PARP1 being the most abundant form, that act as a “molecular nick sensor” to signal SSBs. They play an essential role in mediating the repair of base excisions, being involved in the repair of SSBs 30. Olaparib, a PARP1 inhibitor, blocks this repair effect, leading to the accumulation of SSBs, and these unrepaired SSBs can convert into cytotoxic double‐strand breaks (DSBs) upon cell replication. Under normal circumstances, these DSBs are repaired by means of the potentially error‐free mechanism of the homologous recombination repair pathway 31, key components of which are the tumor suppressor proteins BRCA1 and BRCA2 32. Otherwise, the repair switches to alternative, more error‐prone, mutagenic pathways, such as single‐strand annealing (SSA) and nonhomologous end joining (NHEJ), which are often accompanied by genomic instability, readily resulting in synthetic lethality and cell death 31. Therefore, olaparib shows great advantage in patients with BRCA mutations. As expected, for some cells without these mutations, olaparib, as a single agent, has little effect 11. Our work demonstrated that olaparib alone has little effect on CCA cell lines (TFK‐1, QBC939, HuH28) without BRCA1/2 mutations.

Even though olaparib as a single agent has little effect on tumors without BRCA mutations, the capacity of PARP inhibitors for tumor radiosensitization has been confirmed in a number of *in vitro* and *in vivo* models 24, 33. The theory of selectively exploiting the DNA repair defect from mutations in BRCA1 or BRCA2 by inhibiting another repair pathway is a major breakthrough in the treatment of cancer 20, 34. For cancer cells without BRCA1 or BRCA2 mutations, olaparib can however produce radiosensitization through other mechanisms, which has been reported both *in vivo* and *in vitro* 24, 25. Ionizing radiation induces SSBs, leading to the formation of DSBs at the replication fork; PARP1 is a key enzyme involved in the repair of these, so PARP inhibitors, such as olaparib, could theoretically enhance radiation‐induced tumor cell death. Our data showed that olaparib enhanced the radiosensitivity of CCA cells by inhibiting PARP1. Moreover, we also found that olaparib could enhance the radiosensitivity of immortalized normal cell line *in vitro* (HL‐7702) (Fig. S1), which indicated that olaparib could enhance the radiosensitization of the cells in a high growth condition. While in contrast with normal cells *in vivo*, cancer cells are typically characterized by aberrant cell cycle checkpoint control, defective DNA repair pathways, and accelerated proliferation rates 35. It has been shown that the radiosensitization effect of PARP inhibitors requires DNA replication, so they affect rapidly proliferating tumors more than normal tissues 19.

As a single agent, olaparib has recently been approved for ovarian cancer therapy by the US Food and Drug Administration (FDA) and European Commission in patients with platinum‐sensitive, recurrent, high‐grade serous ovarian cancer with BRCA1 or BRCA2 mutations 36, 37. Many studies have looked at the effect of PARP inhibitors on other solid tumors, as well as ovarian and breast cancer, the first shown to possess the BRCA mutations, including pancreatic and prostate cancer 38, 39. In this study, we first discussed the radiosensitization effect of the PARP inhibitor olaparib in CCA and demonstrated a clear effect, even though there were no significant BRCA mutations in CCA cells. As in previous reports, the degree of radiosensitization depended on the doses of radiation and olaparib, and cell type 11.

These results give us hints that olaparib and radiation combination therapy should be selectively applied to treat CCA. In clinical practice, the recommended dose of olaparib is 400 mg twice daily, making the plasma concentrations of olaparib monotherapy range from 9.29 to 17.19 *μ*mol/L 40. Moreover, it has been reported that the concentration of olaparib in resected breast cancer specimens was on average 41% of the corresponding plasma concentration at the time of surgery 41. Here, we have shown that efficient radiosensitization can be achieved with much lower doses. In spite of the potential for selective killing of tumor cells, our data support the need for a careful phase I dose escalation radiotherapy combination trial. In addition, we should take into consideration the radiation‐induced toxicities with olaparib compared with normal tissue.

In addition, we explored the mechanism of action for the radiosensitization of olaparib by Western blotting and found that radiation‐induced increases in PAR formation may predict for the radiosensitization by PARP1 inhibitor as other study 28. In addition, we found that olaparib and radiation combination treatment increased accumulation of unrepaired DSBs and induced apoptosis in CCA cells.

Our hypothesis proposed a novel strategy to deal with CCA, which is resistant to chemotherapy or radiotherapy. We demonstrated that the combination of the PARP inhibitor olaparib and radiation could contribute to the treatment of CCA, the efficacy being regulated by the radiation dose and the concentration of olaparib. We further showed that the radiosensitization effect of olaparib can be clearly observed at a concentration of 1 *μ*mol/L.

## Conclusion

In conclusion, olaparib shows promise as a radiosensitizer in the clinical treatment of CCA, but its clinical effects and side effects must first be tested. Olaparib produces different degrees of radiosensitization in CCA cells with different genetic backgrounds, which suggests that combination therapy may need to be applied selectively in the clinic owing to genetic diversity among tumors. In addition, optimization of the dosage regimen for radiosensitization is crucial. Genetic diversity is also imperative to develop appropriate diagnostic tests to enable patient selection and identify reliable biomarkers for accurate prognosis with PARP inhibitor therapies.

## Supporting information


**Table S1.** All variants found in next‐generation sequencing for BRCA1 and BRCA2 mutations in QBC939 and TFK‐1 cell lines.

**Figure S1.** The radiosensitization effect of olaparib in HL‐7702 cell line. The viability is normalized to non‐irradiated values at different olaparib doses.
