# Supplementary material for: PARP inhibitor olaparib sensitizes cholangiocarcinoma cells to radiation
Source: Cancer Med. 2018 Feb 26;7(4):1285–96. doi: 10.1002/cam4.1318 (PMC5911590; doi:10.1002/cam4.1318)
Supplement: Supplementary file 1 — Table S1. All variants found in next‐generation sequencing for BRCA1 and BRCA2 mutations in QBC939 and TFK‐1 cell lines. [file CAM4-7-1285-s001.docx]

| Cell lines | Gene | C.dot | P.dot | Genotype | Variant type |  |
| --- | --- | --- | --- | --- | --- | --- |
| QBC939 | BRCA2 | NM_000059.3:c.-26G>A |  | Homozygous | intronic variant | |
|  | BRCA2 | NM_000059.3:c.3396A>G | p.Lys1132= | Homozygous | Synonymous variant | |
|  | BRCA2 | NM_000059.3:c.4068G>A | p.Leu1356= | Homozygous | Synonymous variant | |
|  | BRCA2 | NM_000059.3:c.4563A>G | p.Leu1521= | Homozygous | Synonymous variant | |
|  | BRCA2 | NM_000059.3:c.6513G>C | p.Val2171= | Homozygous | Synonymous variant | |
|  | BRCA2 | NM_000059.3:c.7397T>C | p.Ala2466= | Homozygous | Synonymous variant | |
|  | BRCA1 | NM_007294.3:c.4837A>G | p.Ser1613Gly | Heterozygous | missense variant | |
|  | BRCA1 | NM_007294.3:c.2077G>A | p.Asp693Asn | Heterozygous | missense variant | |
|  | BRCA1 | NM_007294.3:c.3548A>G | p.Lys1183Arg | Heterozygous | missense variant | |
|  | BRCA1 | NM_007294.3:c.3113A>G | p.Glu1038Gly | Heterozygous | missense variant | |
|  | BRCA1 | NM_007294.3:c.2612C>T | p.Pro871Leu | Heterozygous | missense variant | |
|  | BRCA1 | NM_007294.3:c.2311T>C | p.Leu771= | Heterozygous | Synonymous variant | |
|  | BRCA1 | NM_007294.3:c.2082C>T | p.Ser694= | Heterozygous | Synonymous variant | |
|  | BRCA1 | NM_007294.3:c.4308T>C | p.Ser1436= | Heterozygous | Synonymous variant | |
| TFK-1 | BRCA2 | NM_000059.3:c.3396A>G | p.Lys1132= | Heterozygous | Synonymous variant | |
|  | BRCA2 | NM_000059.3:c.3516G>A | p.Ser1172= | Heterozygous | Synonymous variant | |
|  | BRCA2 | NM_000059.3:c.3807T>C | p.Val1269= | Heterozygous | Synonymous variant | |
|  | BRCA2 | NM_000059.3:c.4563A>G | p.Leu1521= | Homozygous | Synonymous variant | |
|  | BRCA2 | NM_000059.3:c.6513G>C | p.Val2171= | Homozygous | Synonymous variant | |
|  | BRCA2 | NM_000059.3:c.7242A>G | p.Ser2414= | Heterozygous | Synonymous variant | |
|  | BRCA2 | NM_000059.3:c.7397T>C | p.Ala2466= | Homozygous | Synonymous variant | |
|  | BRCA2 | NM_000059.3:c.7806-14T>C |  | Heterozygous | intronic variant | |
|  | BRCA2 | NM_000059.3:c.8755-66T>C |  | Heterozygous | intronic variant | |

**Table S1. All variants found in QBC939 and TFK-1 cell lines.**

**Description:**

In TFK-1 cell line,there were seven single nucleotide polymorphisms (SNPs) variants in BRCA2 gene，including seven synonymous variants p.Lys1132= (Heterozygous),p.Ser1172=(Heterozygous),p.Val1269=(Heterozygous), p.Leu1521=(Homozygous),p.Val2171=(Homozygous),p.Ser2414=(Heterozygous),p.Ala2466=(Homozygous), and two intronic variants c.7806-14T>C（Heterozygous）,c.8755-66T>C（Heterozygous）.These variants were classified as Benign based on the frequency of this variant in 1000 Genomes with no effect on splicing or mRNA expression of BRCA2. No variants were in BRCA1. In summay, TFK-1 cell line didn’t have loss of function variants in BRCA1 and BRCA2.

In QBC939 cell line, there were eight heterozygous single nucleotide polymorphisms variants in BRCA1, including three Synonymous variants p.Ser1436=, p.Leu771=, p.Ser694=, five missense variants p.Ser1613Gly, p.Lys1183Arg, p.Glu1038Gly, p.Pro871Leu, p.Asp693Asn.There were six homozygous variants in BRCA2, including five synonymous variants p.Lys1132=, p.Leu1356=, p.Leu1521=, p.Val2171=,p.Ala2466=, and one intronic variant c.-26G>A. These variants were classified as Benign based on the frequency of this variant in 1000 Genomes with no effect on splicing or mRNA expression of BRCA1 and BRCA2. In summay, QBC939 cell line didn’t have loss of function variants in BRCA1 and BRCA2.
