# Supplementary figures and images for: PARP inhibitor olaparib sensitizes cholangiocarcinoma cells to radiation
Source: Cancer Med. 2018 Feb 26;7(4):1285–96. doi: 10.1002/cam4.1318 (PMC5911590; doi:10.1002/cam4.1318)

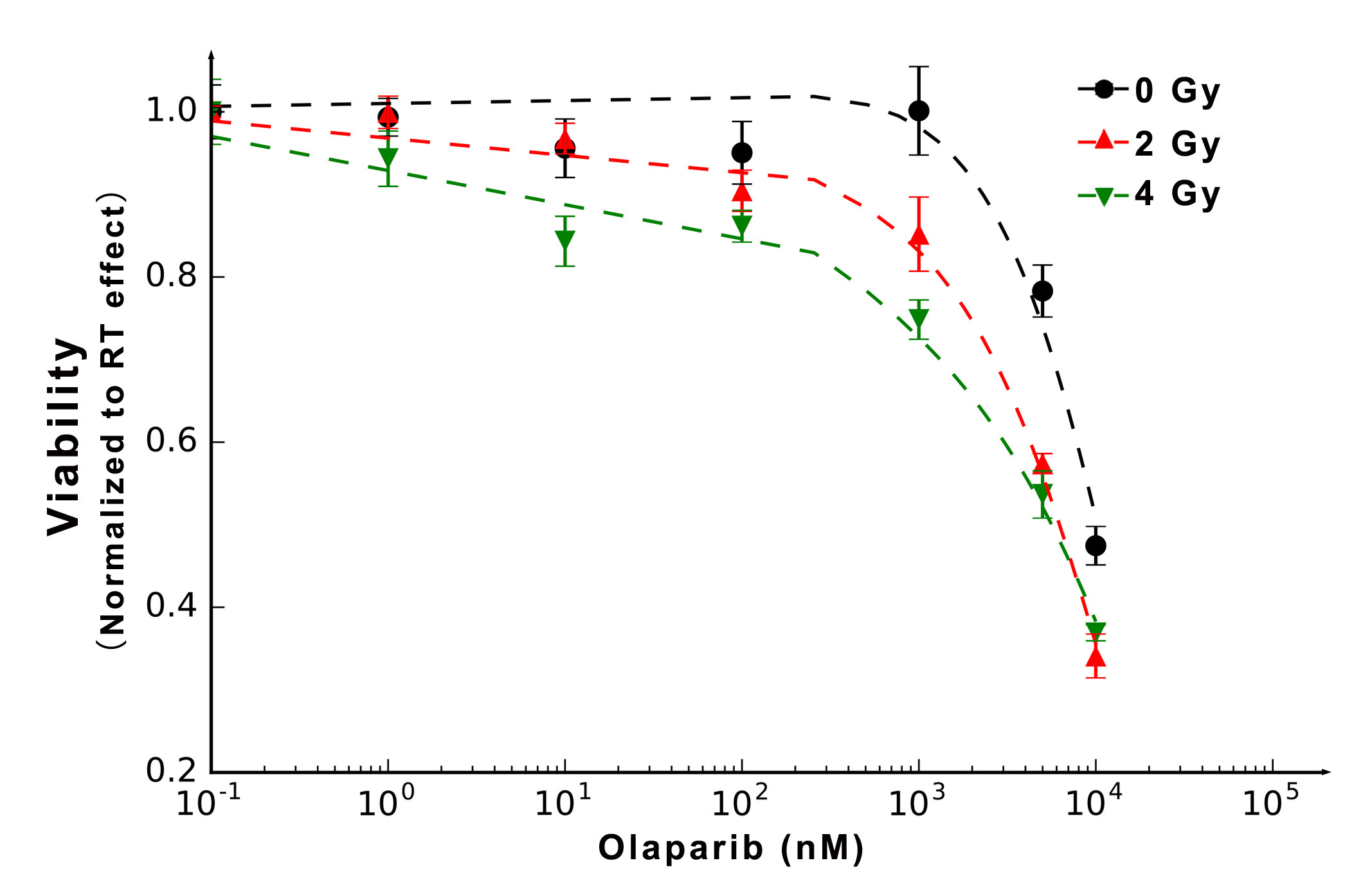

Supplement: Supplementary file 2 — Figure S1. The radiosensitization effect of olaparib in HL‐7702 cell line. The viability is normalized to non‐irradiated values at different olaparib doses. [file CAM4-7-1285-s002.tif]
